# Comparative Analysis of Macro/Microstructures and Constituents of Sorghum and Reed Straw

**DOI:** 10.3390/biomimetics9020107

**Published:** 2024-02-11

**Authors:** Jiafeng Song, Guoyu Li, Yansong Liu, Meng Zou

**Affiliations:** 1 State Key Laboratory of Automotive Safety and Energy, Tsinghua University, Beijing 100084, China; songjiafeng@tsari.tsinghua.edu.cn; 2 Suzhou Automobile Research Institute (Xiangcheng), Tsinghua University, Suzhou 215133, China; 3 Key Laboratory of Transportation Industry for Transport Vehicle Detection, Diagnosis and Maintenance Technology, Jinan 250357, China; 4 School of Mechanical Engineering, Shanghai Dianji University, Shanghai 201306, China; 5 Key Laboratory for Bionics Engineering of Education Ministry, Jilin University, Changchun 130022, China; liuys@jlu.edu.cn (Y.L.); zoumeng@jlu.edu.cn (M.Z.)

**Keywords:** sorghum, reed, cross-section, node characteristics, macro and microstructure, elemental composition

## Abstract

Node-containing straws exhibit superior mechanical properties compared to node-free straw plants, particularly in terms of shear resistance and compression resistance. We explore the relationship between the structure and mechanical properties of straw materials, providing deeper insights for the field of biomechanics. In this study, we focused on two node-containing straw plants, namely sorghum and reed. The main characteristics of sorghum and reed stalks were compared using macroscopic observation, stereomicroscopy, scanning electron microscopy, infrared spectroscopy, and EDS analysis. This study revealed numerous similarities and differences in the macro- and microstructures as well as the elemental composition of sorghum and reed stalks. The functional groups in sorghum and reed stalks were largely similar, with the primary elements being C and O. Distinguishing features included a higher tapering and a slightly larger reduction in wall thickness in sorghum stalks compared to reed stalks. The cross-section of sorghum stalks was filled with pith structures, while reed stalks exhibited a hollow structure. The vascular bundles in sorghum typically showed a paired arrangement, whereas those in reeds were arranged in odd numbers. Furthermore, sorghum straws contained more Cl and no Br, while the parenchyma of reed straws contained higher Br. The C and O proportions of sorghum straws and reed straws are 50–53% (50–51%) and 45–46% (48–49%), respectively. These variations in elemental composition are believed to be correlated with the mechanical properties of the materials. By conducting a detailed study of the micro/macrostructures and material composition of sorghum and reed straw, this paper provides valuable insights for the field of biomechanics.

## 1. Introduction

In nature, there are many biomaterials with unique mechanical properties. Engineers aim to discover these properties and use them to design new structural materials. These materials are thought to possess bionic high-performance properties, including fracture toughness, high specific stiffness, high specific strength, acoustic and vibration isolation, and energy absorption. Biomaterials are an essential source of inspiration for designing new structures, and engineers have realized these new structures in reality. For example, engineers modeled the structure and function of a jellyfish’s ear to create the Jellyfish Ear Storm Predictor [1]. Singapore’s Esplanade Theatre was inspired by durian skins [2]. Beijing’s National Stadium was designed with a bird’s nest in mind [3]. Plant stalks are also a worthy reference for a biological structure.

Plant stalks can be divided into two types: node-containing and node-free types. The slenderness ratios of bamboo, reed, sorghum, and cattail straws can reach 1/100–1/270. Slender straw plants have many stable structures with useful mechanical properties. Their material properties are quite different in different parts of the plant’s stalk [4]. Their structure can be considered a composite material with anisotropic features: the outer layer envelops the pith and nodes [5]. The macrostructure, microstructure, and elemental composition of straws enhance the mechanical properties of straws [6].

Research on the macrostructure of straw mainly focuses on aspects such as the length of the straw and the distribution of fibers in the cross-section [7], which are related to its mechanical properties. Hirai took two flax varieties, Eden and Terre de Lin (TDL), with high and low lodging resistance as research objects, analyzed the relationship between their bending stiffness and fiber distribution, and estimated the modulus of the fibers [8]. Crook found that the lodging resistance of winter wheat significantly differed among different varieties. The lodging resistance of wheat had nothing to do with the strength and stiffness of the stalks, but was mainly related to the length and weight of the stalks [9,10]. The mechanical properties of a rapeseed stem are related to its cross-sectional features [11]. In addition to its macrostructures, its microstructures will also impact its mechanical properties [12].

The microstructures of straws mainly include outer layer tissues, mechanical tissues, vascular bundles, and basic tissues [13]. Liu found significant differences in the stalk cutting characteristics at different growth stages. These differences were mainly due to microscopic differences in the stalks at different growth stages [14]. Kong believed that wheat with a strong lodging resistance had more vascular bundles, a high degree of lignification, and thick mechanical tissues [15]. The number and cross-sectional area of the vascular bundles are related to the mechanical properties of the stalks [16]. The morphology and quantity of the mechanical properties and vascular bundles vary significantly among different pasture varieties, leading to differences in the form and strength of the connections between these tissues [17]. These microscopic differences are the main reasons for the differences in stalk strength and stiffness [18,19]. The study of the mechanical properties of straws must be combined with an internal microstructural characterization to analyze their deformation behavior and mechanism. Due to the complexity of organisms, their chemical compositions also significantly differ between different growth stages [20]. Node straws can be divided into hollow and solid structures. A hollow straw has a hollow structural interior, while a solid straw has obvious inner-core organization. In this study, sorghum and reed, which are typical solid and hollow straws, respectively, were chosen as the research objects.

In addition to this vast diversity in raw materials, natural fibers are also a low-cost, lightweight, and eco-friendly alternative for some synthetic reinforcements, showing acceptable specific properties. The most studied natural plant materials include the stems of cattails, palm trees, coconut trees, and others. Simulating natural structures can lead to the design of lightweight and efficient thin-walled tube structures, enhancing their energy absorption capabilities. Cattails have a hollow multicellular structure that can withstand lateral loads such as wind and rain. In comparison to circular and square tubes, biomimetic multicellular tubes designed based on cattails exhibit better durability in the lateral load direction [21,22]. The node characteristics of palm trees also enhance their bending and compressive strength. Previous studies have applied them to the energy absorption design of biomimetic multicellular carbon fiber-reinforced plastic (CFRP) and aluminum square tubes (Al). Compression tests showed stable progressive folding in the aluminum tubes and progressive end crushing in CFRP tubes. Compared to single-cell tubes, the specific energy absorption (SEA) of double-cell and triple-cell CFRP tubes increased by approximately 17.0% and 2.4%, respectively. The number of cells significantly influences the durability of CFRP tubes and aluminum tubes [23]. Inspired by the structure of a tunicate, a biomimetic conical structure was proposed, comprising an inner conical core structure and an outer-shell framing structure. Through the collaborative action of the outer shell and core structure, the load-bearing capacity of the biomimetic tunicate conical body is greater than the arithmetic sum of the loads borne by the inner core and outer shell. The energy absorption performance of the biomimetic tunicate conical body is also improved under dynamic loading [24].

Until now, there has been limited in-depth research on the macro/microstructures and elemental compositions of sorghum and reed stalks. As cereal crops, sorghum and reed stems have significant potential applications in agriculture and ecology [25,26]. Combined with their natural light weight and high strength, innovative materials can be designed and developed, especially those suitable for research in fields, such as lightweight and efficient thin-walled tubes [27,28].

In this paper, macro/micro- and elemental measurements were used to conduct a comparative analysis of sorghum and reed straws. On the macro scale, measurements were taken of the diameter, wall thickness, internode spacing, and cross-sectional features of sorghum and reed straws. On the micro scale, a comparative analysis was performed on the cross-sectional structure, longitudinal profile, and fiber distribution at nodes of sorghum and reed straws. Regarding the materials, differences in the main elements (C, O) and trace elements (N, K, Ca, Mg, Br) between sorghum and reed straws were analyzed. The macro/microstructures and composition of sorghum and reed straws were studied through comparative analysis, which paved the way for a subsequent mechanical analysis.

## 2. Materials and Methods

### 2.1. Samples

**Figure 1:**
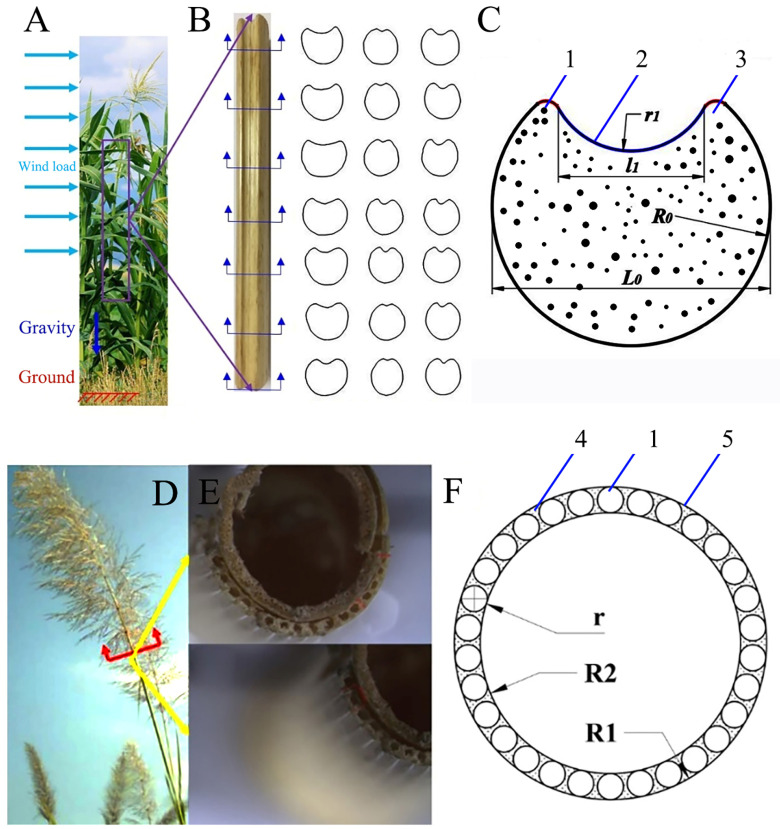
Samples of sorghum and reed straws. (**A**) Sorghum straws, (**B**) cross-section of sorghum straws, (**C**) simplified diagram of sorghum straw cross-section, (**D**) reed straws, (**E**) cross-section of reed straws, (**F**) simplified diagram of reed straw cross-section. 1—vascular bundle, 2—outer layer tissue, 3—basic tissue, 4—thin-walled tissues, 5—mechanical tissue.

Sorghum straws are composite filling structures (Figure 1A). The cross-section of sorghum straws is circular with an oval incision, thus forming a reinforced ring structure similar to a double-ring groove (Figure 1B). Within the stalk are large vascular bundles (which transport water and nutrients) with foamy basal tissues in between. The outer layer tissues are dense and mainly composed of small and dense fibers. The inner pith is a porous foam structure and its function is similar to that of the foam core (Figure 1C). Another common straw with a high length-to-diameter ratio is reed (Figure 1D), which has a hollow stem structure (Figure 1E). It is mainly composed of mechanical tissues, vascular bundles, and thin-walled tissues (Figure 1F). The cross-section of reed straws is mainly composed of an annular hollow structure with diameter *R*_1_ and a unit cell circular structure with diameter r, evenly distributed in the outer layer, and *R*_2_ is the outer diameter of the outer layer.

### 2.2. Observation Positions

The tested macroscopic properties included diameter, wall thickness, node spacing, and cross-sectional morphology. Microstructural characteristics including the transverse cross-section, longitudinal cross-section, and nodes of sorghum and reed straws were observed. Both sorghum and reed straws have high-fiber structures. To ensure the integrity of the cross-sectional structure, the samples were placed under liquid nitrogen freezing for 5 h, and the straws were cut with a sharp blade.

### 2.3. Stereomicroscopy

A stereomicroscope (Stereo, Discovery V20, Oberkochen, Germany) was used here, and its technical parameters were as follows: the total pixels collected by the image analysis system were 10.4 million, the focusing accuracy was 350 nm, and the cold light source was 2150 W.

### 2.4. Scanning Electron Microscopy (SEM) and Energy Dispersive Spectrometer (EDS)

Microscopic research was carried out using a Zeiss scanning electron microscope (SEM, ModelEVO-18, Jena, Germany). The main parameters of SEM were an experimental magnification range of 13–50,000 times magnification, and a minimum resolution of 3.0 nm. Additionally, an energy-dispersive spectrometer (EDS) with a resolution ratio of 6 nm provided the chemical composition of fur samples inside SEM. The elemental composition and contents of the fiber bundles of sorghum, basic tissues of sorghum, mechanical tissues of reed and thin-walled tissues of reed were measured using an energy-dispersive spectrometer (EDS, JSM-5301, Munich, Germany) equipped with SEM.

### 2.5. Infrared Spectra Analysis

The chemical compositions of the sorghum node, sorghum internode, sorghum pith, reed node, and reed internode were tested using a Fourier transform infrared spectrometer (FTIR, NEXUS, Kyoto, Japan). Each sample was crushed into powder with a mortar. Then, 5 mg of the biological sample was mixed with 230 mg of dry potassium bromide, and pressed into a transparent flake with a tablet press. The composition of each sample was detected using a spectrometer (PerkinElmer, Shelton, CT, USA) at a resolution of 4 cm^−1^ in the range of 400–4000 cm^−1^. The background of pure potassium bromide was subtracted from all measured spectra to avoid the influence of water and carbon dioxide.

## 3. Results and Discussion

### 3.1. Comparison of Macrostructures

#### 3.1.1. Changing Measurements of Diameter

**Figure 2:**
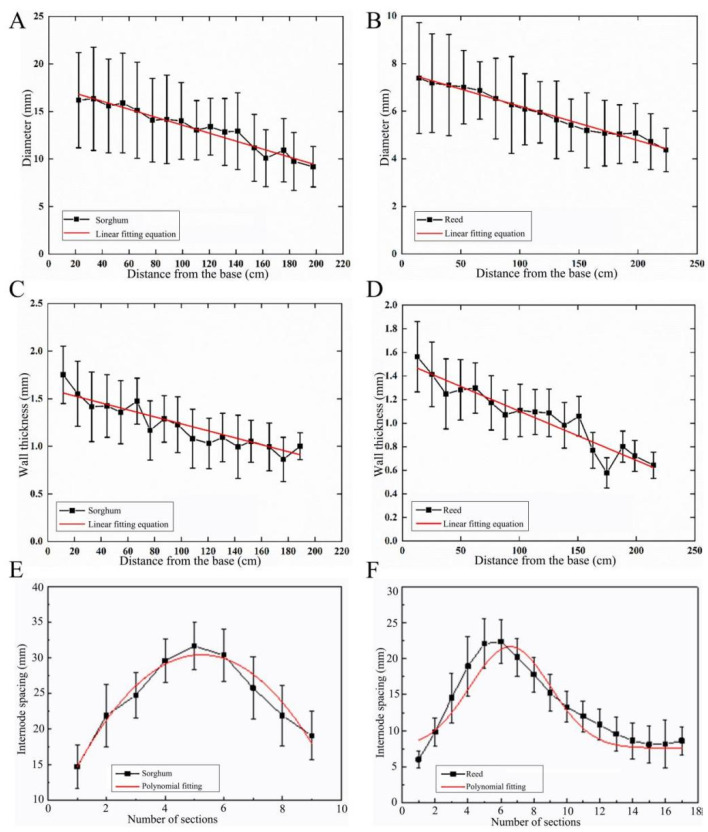
Changes in straw diameter, wall thickness, and internode spacing of sorghum and reed. (**A**) Sorghum straw diameter, (**B**) reed straw diameter, (**C**) sorghum wall thickness, (**D**) reed wall thickness, (**E**) sorghum internode spacing, (**F**) reed internode spacing.

The diameters of sorghum and reed straws were statistically analyzed with respect to their distance from the root (Figure 2A,B). The diameter of the sorghum straws gradually decreased from the root to the top, and was linearly fitted into Equation (1). For the reeds, the changing pattern of their diameter was the same, and was fitted into Equation (2).

(1) $$D_{1}=17.76\;-\;0.042x_{1}\;(R^{2}=0.97)$$

(2) $$D_{2}=7.65\;-\;0.014x_{2}\;(R^{2}=0.98)$$

where *x*_1_ and *x*_2_ are the distance from the roots of sorghum and reed, respectively, in cm, and *D*_1_ and *D*_2_ are the diameters of sorghum and reed, respectively, in mm.

From 20.0 mm from the root to 200.0 mm from the root, the diameters of sorghum and reed decreased from 16.0 mm and 7.5 mm to 9.0 mm and 4.5 mm, respectively. The diameters of the two types of straws gradually decreased from the root to the top at the macroscopic level, indicating they have a conical tubular structure. The difference is that the taper of sorghum straws is higher than that of reed straws. This tapered tubular structure allows the sorghum to withstand greater moments when subjected to lateral loads in nature, such as wind or self-weight.

#### 3.1.2. Wall Thickness Changes

The wall thickness of a reed straw is composed of thin-walled tissues, whereas, for sorghum straws, the wall thickness refers to the outer layer. The wall thickness of the two straws was measured (Figure 2C,D). The changing measurements in both types of straws show a downtrend from root to top. Through linear fitting, the changing measurements of the wall thickness of the two types of straws are expressed in Equations (3) and (4):

(3) $$T_{1}=1.603\;-\;0.004x_{1}\;(R^{2}=0.97)$$

(4) $$T_{2}=1.52\;-\;0.042x_{2}\;(R^{2}=0.96)$$

where *x*_1_ and *x*_2_ are the distance from the roots of sorghum and reed, respectively, in cm, and *T*_1_ and *T*_2_ are the wall thicknesses of sorghum and reed, respectively, in mm.

From 20.0 mm from the root to 200.0 mm from the root, the wall thicknesses of sorghum and reed decreased from 1.5 and 1.4 mm to 1.0 and 0.7 mm, respectively. The changing measurements of the wall thickness of sorghum straws and reed straws are basically the same, except that the wall thickness of sorghum straws decreases slightly more than that of reed straws. The wall thickness decreases in a gradient from the root to the top, promoting a gradient distribution that benefits its load-bearing function.

#### 3.1.3. Changing Measurements of Internode Spacing

Internode spacing is a direct indicator of the node distribution pattern and distribution density. The changing patterns of the internode spacing of the two straws were the same (Figure 2E,F). Polynomial fitting was performed on the measured internode spacing, and the changing measurements can be used in Equations (5) and (6). The overall performance first increased and then decreased.

(5) $$y_{1}=5.44+2.51x_{1}-0.15{x_{1}}^{2}\;(R^{2}=0.97)$$

(6) $$y_{2}=7.59+14.08e^{\left(\frac{-0.5\times ({x_{2}}^{2}-6.56)}{2.45}\right)}\;(R^{2}=0.96)$$

where *x*_1_ and *x*_2_ are the order of nodes from the root to the top of sorghum and reed, respectively, and *y*_1_ and *y*_2_ are the internode spacing of sorghum and reed, respectively, cm.

The maximum and minimum internode spacings of sorghum and reed straws are located at the 6th and 1st nodes, respectively. The maximum and minimum internode spacings are 32.0 and 15.0 mm in sorghum, respectively, and 22.5 and 6.0 mm in reed, respectively. The changing measurements of the node characteristics of the two straws are sparse in the middle and dense on both sides, and the density of the nodes at the root is slightly higher than at the top. More nodes can effectively increase the strength of straws, thereby ensuring that the straws do not lodge.

#### 3.1.4. Cross-Sectional Properties

**Figure 3:**
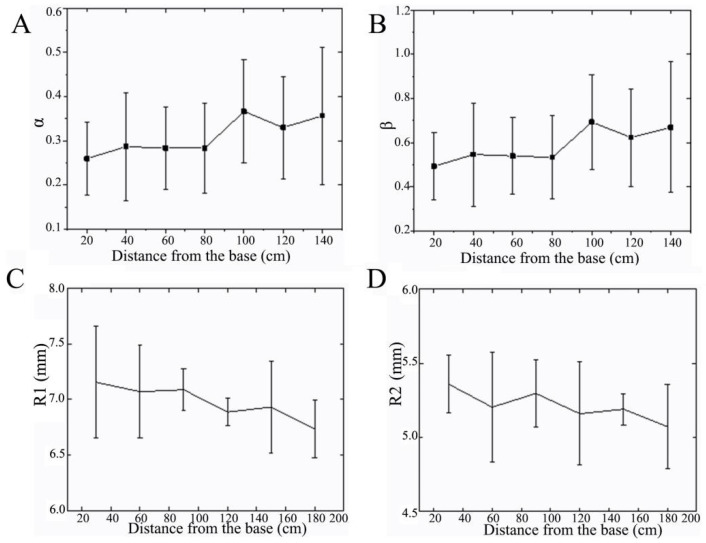
Cross-section parameters of sorghum and reed straws. (**A**,**B**) *α* and *β* of sorghum straws, (**C**,**D**) *R*_1_, *R*_2_, and *r* of reed straws.

The structure of the cross-section plays an important role in the bending behavior of the entire rod [29,30]. Sorghum straws mainly bear wind loads and gravity, which is similar to cantilever beams in the engineering field. A sorghum straw has a circular cross-section with arc grooves. The circular groove structure is mainly determined by two parameters (Figure 3A,B). *α* is the ratio of *l*_1_ (the width of the groove) to *L*_0_ (the sectional diameter) (*α* = *l*_1_*/L*_0_), and measures the width of the groove. This ratio affects the shape of the cross-section and the straw’s wind load carrying capacity. *β* is the ratio of *r*_1_ (the radius of the groove) to *R*_0_ (the sectional radius) (*β* = *r*_1_*/R*_0_), and measures the depth (or curvature) of the groove. This ratio affects the curvature of the cross-section and the stiffness of the overall structure. By analyzing the values of *α* and *β*, the structure of sorghum straws can be optimized to better resist wind load and gravity, improve the bending performance of the entire rod, and guide the bionic design of thin-walled tubes.

The cross-section of reed straws is mainly composed of *R*_1_ and *R*_2_, where *R*_1_ is the outer diameter of the mechanical tissue, and *R*_2_ is the inner diameter of the mechanical tissue. The mechanical tissue of reed straws also appears to be a tapered tubular structure from root to top (Figure 3C,D).

### 3.2. Comparison of Microstructures

#### 3.2.1. Structural Analysis under a Microscope

**Figure 4:**
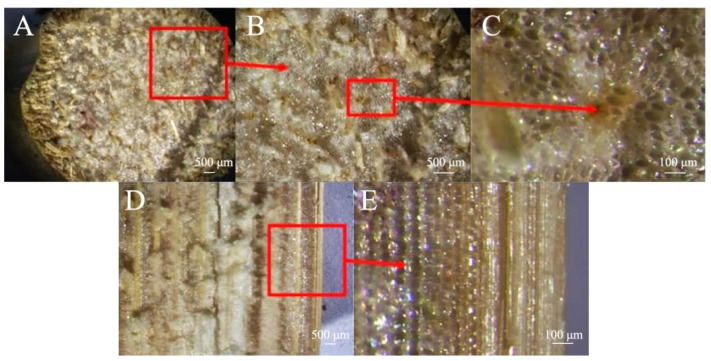
Stereomicroscopic view of sorghum straw. (**A**,**B**) Transverse section, (**C**) vascular bundle structure, (**D**) longitudinal section, (**E**) cavity structures.

Since the epidermal cell wall of sorghum contains silicon salt, the hardness of the outer layer is relatively high [31,32]. The transverse cross-section of sorghum straws is clearly filled with pith structures (Figure 4A,B). Vascular bundle structures of different sizes are distributed among them, and the vascular bundles are mostly distributed vertically (Figure 4C). Longitudinally, the vascular bundles are not a complete canal structure (Figure 4D), and cavity structures of different sizes are distributed within the vascular bundles (Figure 4E).

**Figure 5:**
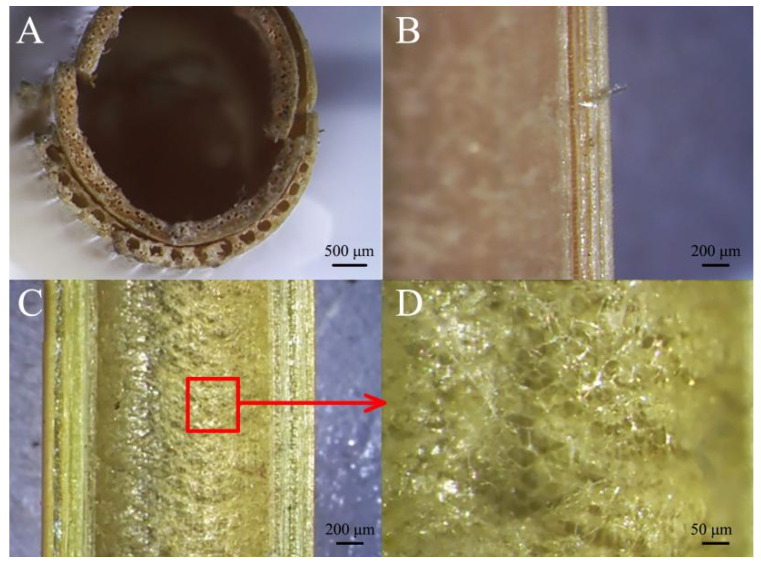
Stereomicroscopic view of reed straw. (**A**) Transverse section, (**B**) longitudinal section of vascular bundles, (**C**) inner wall structure, (**D**) inner wall of thin-walled tissue.

Reed straws are different from sorghum straws. The mechanical tissue of reed is characterized by the presence of similarly sized circular hole structures (Figure 5A). The longitudinal view of the vascular bundles of reed straws is similar to that of sorghum straws, as there are cavity structures of different sizes (Figure 5B). There is no completely filled pith structure inside the reed straws (Figure 5C). On the inner wall of the thin-walled tissue is a spider-web-like pulp structure (Figure 5D).

#### 3.2.2. Comparison of Microscopic Transverse Cross-Sections

Although stereomicroscopy can reveal the microscopic structural characteristics of samples through magnified observation, it is still unable to clearly observe the microstructures of the samples. Therefore, on the basis of stereomicroscopic observation, the micromorphological characteristics of the two types of straws were observed through SEM.

**Figure 6:**
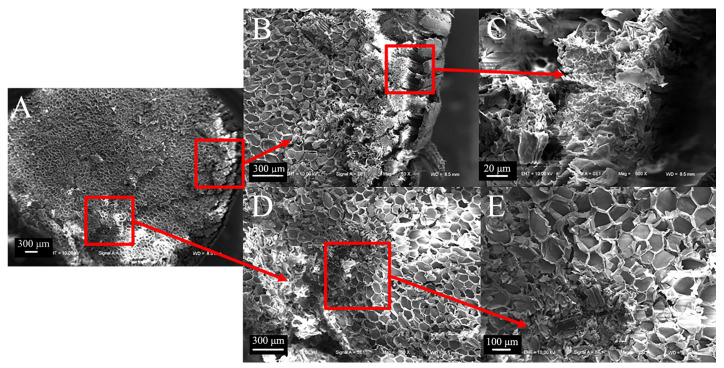
Transverse electron microscope view of sorghum straws. (**A**) Transverse section of sorghum (50×), (**B**) outer layer of sorghum (100×), (**C**) partial view of outer layer of sorghum (800×), (**D**) basic tissue combined with vascular bundle of sorghum (100×), (**E**) partial view of vascular bundle of sorghum (200×).

The cross-section of sorghum straw exhibits a substantial distribution of vascular bundles (Figure 6A). Stratified structures distributed on the outer layer are tightly arranged, without cavities (Figure 6B,C). The vascular bundles are dispersed vertically within the basic tissues, forming a radial pattern. From the outer to the inner regions, the number of vascular bundles gradually decreases, leading to reduced density. However, the area of the cavities within the vascular bundles increases (Figure 6D). In sorghum (Figure 6E), the vascular bundles often exhibit paired arrangements (combinations of two large bundles or two small bundles). The vascular bundles of the basic tissues are mainly elliptical, resulting in a discontinuity in the cross-section of sorghum straws in that plane. Their spatial arrangement exhibits directionality, imparting anisotropic properties to the material.

**Figure 7:**
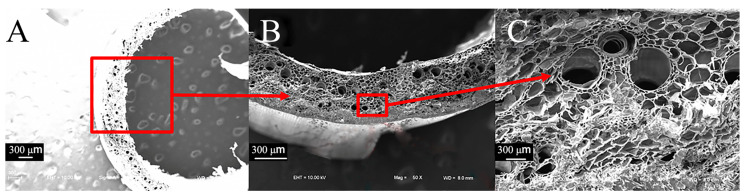
Transverse electron microscope view of reed straws. (**A**) Transverse section of reed (50×), (**B**) thin-walled tissue combined with vascular bundle of reed (100×), (**C**) detailed view of vascular bundle of reed (200×).

Compared to sorghum straws, reed straws have a hollow structure consisting mainly of mechanical tissue and thin-walled tissue. The thin-walled tissue contains cavities and vascular bundles (Figure 7A,B) arranged in an odd-numbered combination, typically with two large vascular bundles and one small vascular bundle (Figure 7C).

#### 3.2.3. Comparison of Microscopic Longitudinal Cross-Sections

**Figure 8:**
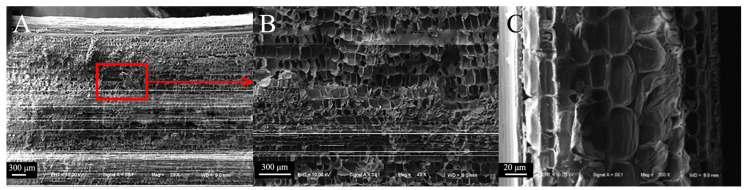
Longitudinal electron microscope view of sorghum and reed straws. (**A**) Longitudinal section of sorghum (100×), (**B**) Basic unit cell structure of vascular bundles in sorghum (100×), (**C**) basic unit cell structure of vascular bundles in reed (800×).

From the longitudinal cross-section, it can be observed more intuitively that the epidermal tissues of sorghum straws are closely bound without gaps (Figure 8A), while their basic tissues are contain cavities of various sizes (Figure 8B). The thin-walled tissue of reed also contains cavities of varying sizes, with the cavities becoming smaller closer to the mechanical tissue (Figure 8C). The outer layer tissue of sorghum straw can enhance its shear and compression resistance.

#### 3.2.4. Comparison of Microscopic Node Characteristics

**Figure 9:**
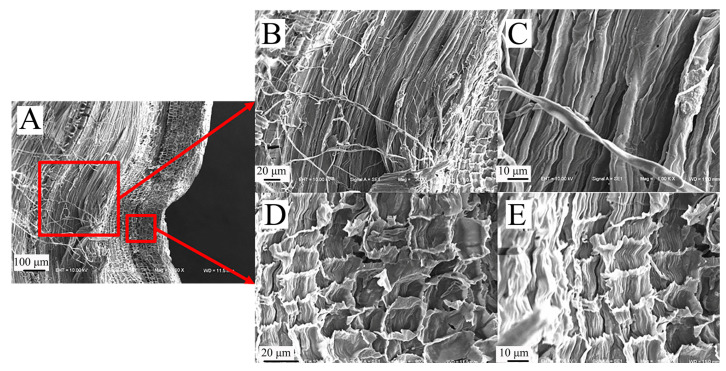
Microscopic node characteristics of sorghum straws. (**A**) Transverse section of sorghum nodes (200×), (**B**) sorghum fiber layer of sorghum nodes (800×), (**C**) partial view of fiber layer (1600×), (**D**) cavity structures of sorghum nodes (800×), (**E**) partial view of cavity structures (1600×).

The fibers of the epidermal tissue of sorghum straws bend and fold outward at the nodes (Figure 9A), and fiber fractures occur at the nodes (Figure 9B,C). Compared to the vascular bundles between nodes, the vascular bundle cavities at the nodes are smaller and more densely distributed (Figure 9D,E). This dense fiber structure can increase the shear and compression resistance of sorghum straw.

**Figure 10:**
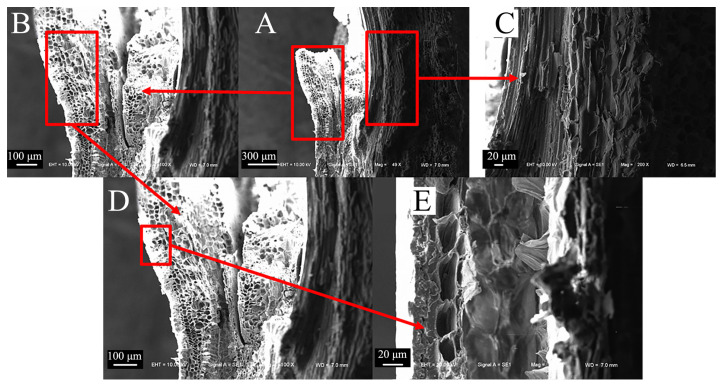
Microscopic node characteristics of reed straws. (**A**) Transverse section of reed nodes (200×), (**B**) thin-walled tissue of reed nodes (100×), (**C**) mechanical tissue of reed nodes (800×), (**D**) partial view of thin-walled tissue (200×), (**E**) cavity structures of thin-walled tissue (800×).

The microscopic node features of reed share many similarities with sorghum (Figure 10A). For instance, the distribution of the cavities in the thin-walled tissue is relatively dense (Figure 10B–E), and the fibers in the mechanical tissue of reed also bend outward and fold, and are arranged closely (Figure 10C).

### 3.3. Comparison of Chemical Composition and Functional Groups

#### 3.3.1. Functional Groups

**Figure 11:**
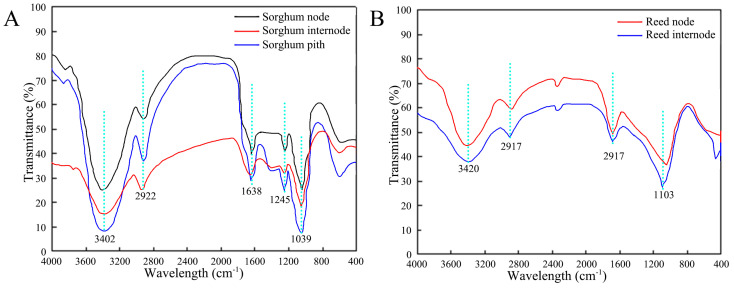
FTIR spectra of straws. (**A**) Sorghum straw, (**B**) reed straw.

The infrared spectra of sorghum and reed samples were measured at different locations, including the sorghum node, sorghum internode, sorghum pith (Figure 11A), reed node, and reed internode (Figure 11B). The infrared spectral curves of the two types of straws are similar, and the positions of their absorption peaks are basically the same, indicating that the types of functional groups contained in each part are almost the same. However, the values of the peaks at the same position differ, indicating that the relative contents of the functional groups contained in each part are different.

The spectra of the straws conform to typical plant fiber constituents, and the functional groups of sorghum and reed straw are not different. The broad and strong peaks at 3402 cm^−1^ in sorghum and 3420 cm^−1^ in reed are mainly the stretching vibration absorption caused by the hydroxyl group (-OH) in cellulose and hemicellulose. The peaks are wide due to the stretching vibration of the associated hydrogen bonds (-OH···O). The peaks at 2922 cm^−1^ in sorghum and 2917 cm^−1^ in reed are caused by the antisymmetric stretching vibration of saturated hydrocarbons in cellulose and hemicellulose and the stretching vibration of -CH. The peak at 1245 cm^−1^ in sorghum and 1250 cm^−1^ in reed are caused by the vibration of CO in hemicellulose [33,34]. The peak at 1039 cm^−1^ in sorghum and 1103 cm^−1^ in reed are ascribed to the vibration of CO in cellulose. Infrared spectroscopy can only qualitatively analyze whether the straw contains functional groups. Therefore, this paper continues to use energy spectrum analysis to quantitatively analyze the elemental contents of the straw.

#### 3.3.2. Element Composition

The main elements in the fiber bundles, in the basic tissue of sorghum straws, and in the mechanical tissues and parenchyma of reed straws are shown in Table 1. Sorghum and reed have similar main elements, both containing carbon (C) and oxygen (O). For sorghum straws, the C content is the highest (40–53%), followed by O (45–46%). For reed straws, the C content is the highest (50–51%), followed by O (48–49%). In terms of trace elements, sorghum straws contain more Cl and no Br, while the parenchyma of reed straws contains more Br. In addition, both sorghum straws and reed straws contain additional K. These variations in elemental composition are believed to be correlated with shear and compression resistance. The different types and proportions of the chemical elements in sorghum straws and reed straws will lead to differences in their hardnesses and elastic moduli, further affecting their mechanical properties.

## 4. Conclusions

This paper employs macro/micro- and elemental measurements to analyze the macro/microstructures and composition of sorghum and reed straws, laying the foundation for their subsequent mechanical analysis. This study reveals both similarities and differences between sorghum and reed straws in terms of their macro/microstructures and elemental composition.

In macroscopic terms, both straws exhibit a conical tube structure. From 20.0 mm to 200.0 mm from the root, both sorghum and reed straws show a reduction in diameter and wall thickness. The node characteristics in both straws follow a pattern of being sparse in the middle and denser on both sides, with a slightly higher density at the root compared to the top. Increased numbers of nodes effectively enhance straw strength, preventing lodging. At the microscopic level, both sorghum’s basic tissue and reed’s thin-walled tissue feature numerous vertically distributed vascular bundles surrounded by unevenly sized cavities. From the outer to the inner regions, the number of vascular bundles decreases, leading to reduced density, while the area of the cavities within the vascular bundles increases. Sorghum’s outer epidermal tissue and reed’s mechanical tissue exhibit a layer-like structure without cavities. At the nodes, both sorghum’s epidermal tissue and reed’s mechanical tissue bend outward, and the vascular bundle cavities at the nodes are smaller and more densely distributed. In terms of elemental composition, sorghum and reed straws share basic functional groups, with carbon (C) and oxygen (O) being the main elements.

Macroscopically, sorghum straws exhibit a higher taper than reed straws, allowing them to withstand greater lateral loads in nature. The decrease in wall thickness from the root to the top is slightly greater in sorghum straws. This decreasing gradient of wall thickness is favorable for its load-bearing function. Microscopically, sorghum straws have a medullary structure in their cross-section, while reed straws have a hollow structure with a spider-web-like pith on the inner wall of their thin-walled tissues. Reed mechanical tissue features regular-sized circular hole structures. Sorghum’s vascular bundles typically align in pairs, while reed’s vascular bundles exhibit an odd-numbered arrangement. In terms of elemental composition, sorghum and reed straws differ. Sorghum contains more chlorine (Cl) and no bromine (Br), while reed straws contain higher levels of Br in their thin-walled tissue.

The macro/microstructures and elemental composition of sorghum and reed directly affect their mechanical properties, including their tensile strength, rigidity, and flexibility. In-depth research on these structures and elements helps us to understand the properties of these natural materials and provides references for their rational utilization in biomedical engineering or the biomimetic applications of thin-walled tubes.

## Figures and Tables

**Table 1:** Contents and proportions of the main elements in sorghum and reed.

Element	C	O	Cl	K	Ca	Mg	Br
Fiber bundles of sorghum	53	45	1	1	0	0	0
Basic tissues of sorghum	50	46	1	1	1	1	0
Mechanical tissues of reed	51	49	0	0	0	0	0
Thin-walled tissues of reed	51	48	0	0	0	0	1

## Data Availability

The raw data supporting the conclusions of this article will be made available by the authors without undue reservation.
